# The Relationship between Social Isolation and Sense of Community among Older Adults in Puerto Rico

**DOI:** 10.1093/geroni/igab046.3173

**Published:** 2021-12-17

**Authors:** Thomas Buckley, Denise Burnette

**Affiliations:** 1 Virginia Commonwealth University, NORFOLK, Virginia, United States; 2 Virginia Commonwealth University, Virginia Commonwealth University, Virginia, United States

## Abstract

Psychological sense of community (SOC) is linked to key health and wellbeing outcomes for older adults and among Latin American populations. Prior research shows that social factors may affect SOC, but this has yet to be studied among Puerto Rican older adults. This study draws on Social Resource Theory to test the hypothesis that social isolation is associated with SOC among older adults in Puerto Rico. We collected data through face-to-face interviews in a non-probability sample of community dwelling adults aged 60+ throughout Puerto Rico in 2019-2020 (N = 154). We measured social isolation with the Spanish translation of the LSNS-6 (range 0-30, mean= 14.00, SD= 5.99), where higher scores indicate less isolation, and SOC with the Spanish translation of the Brief Sense of Community Scale (range 0-32, mean= 24.75, SD= 6.04). This cross-sectional study used multiple linear regression to test the association between social isolation and SOC, while controlling for gender, age, income and living arrangement. Higher scores on the LSNS-6 were associated with higher SOC (β=0.31, SE=0.08, p<0.001). Among the sociodemographic covariates, increased age was associated with higher SOC (β=0.12, SE=0.05, p<0.05). This study demonstrates that older adults in Puerto Rico who are more socially isolated have lower SOC, and that SOC increases with age. In order to promote SOC in this population, interventions should focus on reducing social isolation and may benefit from targeting young-old older adults. Future research should continue to examine these relationships and extend to other Latin American cultures.

